# Functional connectivity supporting the selective maintenance of feature-location binding in visual working memory

**DOI:** 10.3389/fpsyg.2014.00507

**Published:** 2014-06-02

**Authors:** Sachiko Takahama, Jun Saiki

**Affiliations:** Graduate School of Human and Environmental Studies, Kyoto UniversityKyoto, Japan

**Keywords:** dorsolateral prefrontal cortex, feature-location binding, functional connectivity, hippocampus, maintenance

## Abstract

Information on an object's features bound to its location is very important for maintaining object representations in visual working memory. Interactions with dynamic multi-dimensional objects in an external environment require complex cognitive control, including the selective maintenance of feature-location binding. Here, we used event-related functional magnetic resonance imaging to investigate brain activity and functional connectivity related to the maintenance of complex feature-location binding. Participants were required to detect task-relevant changes in feature-location binding between objects defined by color, orientation, and location. We compared a complex binding task requiring complex feature-location binding (color-orientation-location) with a simple binding task in which simple feature-location binding, such as color-location, was task-relevant and the other feature was task-irrelevant. Univariate analyses showed that the dorsolateral prefrontal cortex (DLPFC), hippocampus, and frontoparietal network were activated during the maintenance of complex feature-location binding. Functional connectivity analyses indicated cooperation between the inferior precentral sulcus (infPreCS), DLPFC, and hippocampus during the maintenance of complex feature-location binding. In contrast, the connectivity for the spatial updating of simple feature-location binding determined by reanalyzing the data from Takahama et al. (2010) demonstrated that the superior parietal lobule (SPL) cooperated with the DLPFC and hippocampus. These results suggest that the connectivity for complex feature-location binding does not simply reflect general memory load and that the DLPFC and hippocampus flexibly modulate the dorsal frontoparietal network, depending on the task requirements, with the infPreCS involved in the maintenance of complex feature-location binding and the SPL involved in the spatial updating of simple feature-location binding.

## Introduction

Visual working memory (VWM) plays an important role in the maintenance of multi-dimensional object representations that are no longer present in the environment. VWM suffers from severe capacity limitations (Kahneman et al., 1992; Luck and Vogel, 1997; Rensink, 2000; Cowan, 2001; Wheeler and Treisman, 2002). Thus, an important question is how the visual system manages to maintain only the information that is necessary for efficient interactions with the world. One question in this selective maintenance problem is whether observers can selectively maintain task-relevant feature combinations among multi-dimensional objects. The current study investigated the brain regions and functional connectivity underlying the selective maintenance of feature-location binding.

Previous studies are equivocal on whether observers can selectively maintain task-relevant feature-location binding (Luck and Vogel, 1997; Wheeler and Treisman, 2002; Treisman and Zhang, 2006). Luck and Vogel (1997) have shown that people can store a fixed number of objects in VWM regardless of the number of task-relevant dimensions. Wheeler and Treisman (2002) have reported some costs of the maintenance of feature-location binding, and Treisman and Zhang (2006) have shown interference by task-irrelevant features, suggesting that the selective maintenance involves certain costs. One possible factor that contributes to this inconsistency is the role of location information in VWM. Some studies have indicated special roles of location in VWM (Kahneman et al., 1992; Kondo and Saiki, 2012). Kondo and Saiki (2012) have shown that interference occurs only when location information is task-irrelevant. Many previous studies that have reported the costs and interferences contain comparisons between location-relevant and location-irrelevant conditions, and, thus, their results could be accounted for by the role of location. Therefore, in order to investigate the selective maintenance of feature-location binding in a general sense, we should use a task in which location is always task-relevant. The current study accomplished this by using a stimulus set composed of color, orientation, and location. With identical stimuli, one task required the maintenance of a triple conjunction (color-orientation-location), which is called the complex feature-location binding task, and the other task required the maintenance of a single conjunction (color-location or orientation-location), which is called the simple feature-location binding task. This paradigm enabled us to examine the effects of the complexity of feature-location binding in VWM without a confound involving the role of location.

Previous neuroimaging studies of VWM tasks have indicated the involvement of the dorsolateral prefrontal cortex (DLPFC) (Linden et al., 2003; Mohr et al., 2006; Axmacher et al., 2007; Jackson et al., 2011) and hippocampus (Piekema et al., 2006; Axmacher et al., 2007; Hannula and Ranganath, 2008; Howard et al., 2011) and have examined functional connectivity (Rissman et al., 2008; van Vugt et al., 2010; Santangelo and Macaluso, 2013). Previous studies focusing on feature-location binding have shown the involvement of the parietal cortex (Corbetta et al., 1995; Wojciulik and Kanwisher, 1999; Shafritz et al., 2002; Todd and Marois, 2004; Xu and Chun, 2006) and anterior frontal lobule (Mitchell et al., 2000; Prabhakaran et al., 2000). In the current study, we used event-related functional magnetic resonance imaging (fMRI) to assess the role of these regions and the functional connectivity for the maintenance of complex feature-location binding compared to simple feature-location binding.

If a brain region is affected by the effects of the complexity of feature-location binding, then the brain region would show activation that was increased in the complex feature-location binding condition compared to that in the simple feature-location binding condition. It is important to test whether the observed effects of complex feature-location binding merely reflect general memory load. For this purpose, we compared the neural networks for complex feature-location binding with those for the spatial updating of simple feature-location binding by reanalyzing the data of a previous study (Takahama et al., 2010). Both the current study and Takahama et al. (2010) utilized a paradigm called multiple object permanence tracking (MOPT; Saiki, 2003), which can simultaneously investigate both feature-location binding and its spatial updating of object representation. Brain activation and functional connectivity that is specific to complex feature-location binding would likely show a pattern that is distinct from that observed in the spatial updating task.

## Materials and methods

### Participants

Twenty-two healthy volunteers (18 males, 4 females; mean age = 26.5 years old) participated in the current study. All participants were healthy with normal or corrected-to-normal vision and visual acuity and no history of neurological or psychiatric episodes. They provided informed written consents prior to experimentation, in accordance with the research ethics committee of the Graduate School of Human and Environmental Studies, Kyoto University. Twenty of 22 participants were right-handed.

### Stimulus display

**Figure 1:**
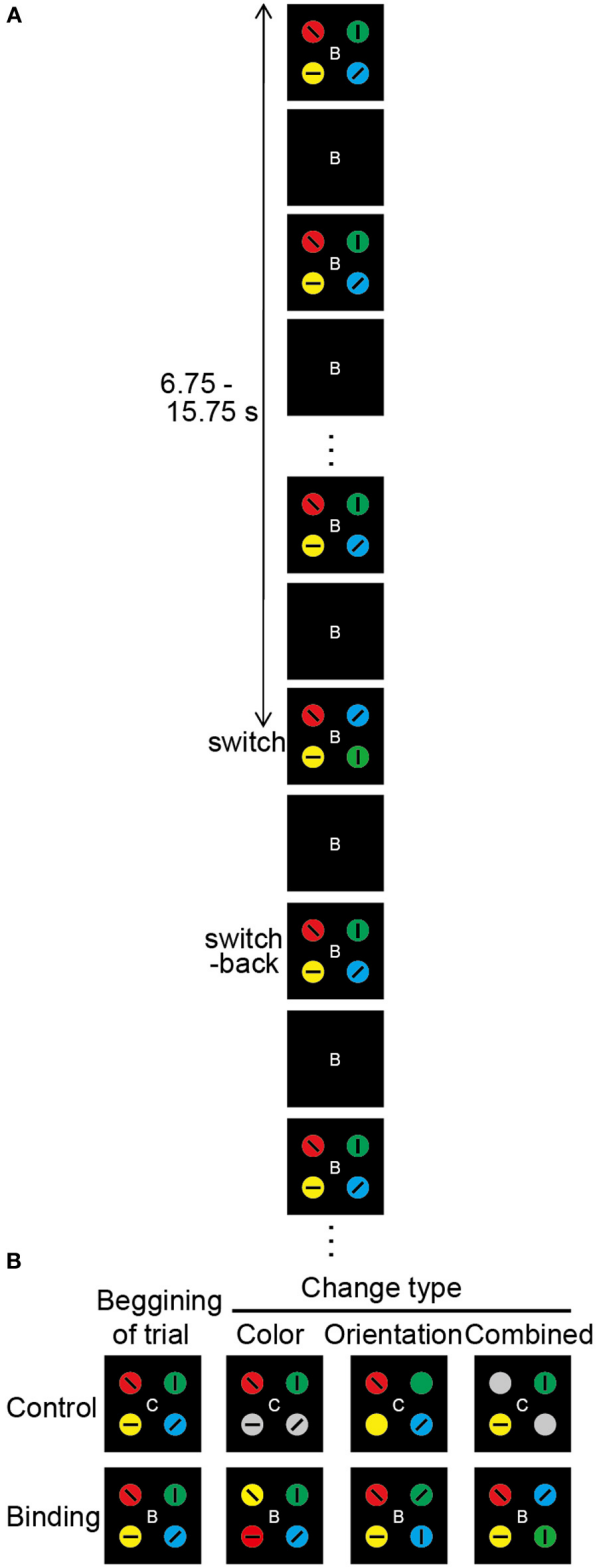
**Overview of the change detection task. (A)** Schematic diagram of the change detection task (combined binding change). At the beginning of each trial, the occluder, and the capitalized letter (B or C) at the center of the visual stimuli are presented for 500 ms. Then, the occluder begins to move. The targets gradually appear, become gradually occluded, and gradually reappear, which repeats until the end of the trial. The direction of occluder motion (clockwise or anticlockwise), as well as the timing of the changes in visual stimuli, is randomly varied across trials. The durations of the target-visible and target-invisible periods are identical (375 ms). The change in targets occurs during a single visible period, with each trial lasting 20 s. Altered colors and/or orientations are switched back during the next visible period. After a 1.5-s inter-trial interval, the occluder and the capital letter for the next trial appear. In each task, participants are asked to push a response button when they notice a task-related change. **(B)** Types of target change. The change in the simple feature-location binding corresponds to color or orientation change, whereas the change in the complex feature-location binding corresponds to combined change. In the color-control change, 2 of 4 color targets turn gray. In the color-binding change, the colors are switched for 2 of 4 targets. In the orientation-control change, 2 of 4 tilted bars disappear. In the orientation-binding change, the tilted bars are switched for 2 of 4 targets. In the combined control change, the colors turn gray and the tilted bars disappear for 2 of 4 targets. In the combined binding change, the tilted bars and embedded colored circles of two objects are simultaneously replaced. In the color task, the types of task-relevant changes are color-control, color-binding, combined control, and combined binding. In the orientation task, the types of task-relevant changes are orientation-control, orientation-binding, combined control, and combined binding. In the conjunction task, the combined control and combined binding are task-relevant change.

To test the selective maintenance of task-relevant feature-location binding, a stimulus display composed of four objects was utilized; each object was defined by a combination of color and orientation. In addition, each object comprised a tilted black bar that was embedded in a colored circle with four objects configured in a radial pattern. On top of the pattern, a windmill-shaped occluder rotated at a constant angular velocity (126°/s) so that the pattern of the objects repeatedly appeared and disappeared (Figure 1A). Participants monitored the changes in the object features across the visible period and reported a change as quickly as possible if it occurred. The durations of the visible and occluded periods were identical (375 ms). The direction of occluder rotation (clockwise or counterclockwise) was randomly determined across trials. Each circle (1.65°) and tilted bar (1.24 × 0.62°) was placed at a visual angle of 3.42° from the center of the occluder. The target object colors were red, blue, yellow, green, purple, orange, or pink. The orientations of the tilted black bars were 0, 45, 90, or 135°. For each stimulus display, four objects were always presented with different colors and orientations.

In each trial, only one visible period contained a change in object features; the objects changed during one visible period but returned to the original state in the next visible period. The timing of the visual changes varied across trials from 6.75 to 15.75 s after sequence onset, and it was therefore unpredictable for participants. Six different types of change events were created by the manipulation of two factors: switch type (binding/control) and changed feature (color/orientation/combined) (Figure 1B). The simple feature-location binding corresponded to color-location or orientation-location binding, whereas the complex feature-location binding corresponded to color-orientation-location binding. Changes in the binding condition were implemented by switching the feature values of two objects. Thus, the maintenance of feature-location binding was necessary for detecting the changes. The changed feature was either color alone, orientation alone, or both (combined). In the color-binding and orientation-binding change trials, the colors and orientations, respectively, of two objects alternated between each other during one visible period. Following the occlusion, the features returned to the initial configuration. In the combined-binding change trials, both the color and the orientation of the two objects were simultaneously switched during one visible period.

In the control condition, it was possible to detect a change event without consideration of the feature combinations. To equate the physical amount of change with the binding condition, the changes occurred for two objects. In the color-control change trials, the two objects turned gray. In the orientation-control change trials, the embedded bars for two objects disappeared. In the combined-control change trials, the color-control, and orientation-control changes occurred simultaneously in the two objects. Every possible combination of two objects (e.g., upper left and lower left, upper left and upper right) from among the four display elements was targeted for a feature-location change occurring equally often across trials. Therefore, the participants had to monitor all four objects. To alert participants to the switch type involved in the change event, a capitalized letter (“C” for control or “B” for binding) was presented at the display center throughout each trial (the letter was shown for 500 ms before moving the display). The stimulus presentation and response measurements were controlled with a computer with a Windows operating system and a visual stimulus generator (ViSaGe; Cambridge Research Systems Ltd., Rochester, Kent, UK) in conjunction with Matlab (The MathWorks, Inc., Natick, MA, USA).

### Change detection task

The task relevance was based on the instructions administered prior to a given run. To determine the impact of the selective maintenance of feature-location binding, three different change detection tasks were utilized by the manipulation of task-relevant feature-location binding together with identical stimulus displays. The three tasks were subdivided into two major classes: the simple and complex feature-location binding tasks. In the simple feature-location binding task, participants were asked to monitor simple feature-location binding, such that one feature was bound to a location, while the other feature was task-irrelevant. There were two types of simple feature-location binding tasks: color task and orientation task. In the color and orientation tasks, the task-relevant change events were the color and combined events and the orientation and combined events, respectively. In the complex feature-location binding task, which was referred to as the conjunction task, participants monitored complex feature-location binding, such that both color and orientation were bound to location. In this case, the only task-relevant change event was the combined change.

Participants were instructed to press a response button when they detected a task-relevant change. In the simple change detection paradigm, participants simply reported the presence of a change. Thus, it did not matter what type of change had been made. However, the task-relevant change detection paradigm required that participants distinguish between the different types of changes and respond selectively based on the instructional manipulation. A correct response was defined as a response during the change period (375 ms) or during the occluded period (375 ms) immediately after the change period in the task-relevant change trial as well as no response in the task-irrelevant change trial. An error was considered a failure to respond to the task-relevant change or responses that occurred during an unchanged stimulus display in the task-relevant change trial as well as any response in the task-irrelevant change trial. In the current study, a typical error was considered a failure to respond to the task-relevant change.

The three change detection tasks (color, orientation, and conjunction) were conducted in 3 fMRI runs, respectively. The simple feature-location binding tasks (color and orientation) preceded the complex feature-location binding task (conjunction), and the order of the simple feature-location binding tasks was counterbalanced across participants. At the beginning of each run, participants received the instructions for the task to be performed. There were 25 trials within each run (13 control and 12 binding trials), lasting 22 s each. The binding condition trials were interleaved with the control trials. All change types were presented in a randomized order separately in the control and binding conditions. In all three tasks, the task-relevant change occurred in 66.7% of the trials, which indicated that the proportion of color, orientation, and both change trials differed between the simple and complex feature-location binding tasks.

Prior to the scanning session, participants underwent 1–5 practice runs for each change detection task outside of the scanner until a criterion performance was achieved (>80% accuracy in all tasks). The same stimuli were later used during scanning. To avoid verbal encoding, articulatory suppression was utilized during the practice runs. Participants were asked to repeatedly vocalize “da, da, da…” while a stimulus sequence was shown. However, the articulatory suppression was not used in the fMRI sessions. Participants subsequently completed an additional practice run inside the scanner immediately prior to the fMRI sessions. After the fMRI sessions, they reported that they did not verbalize the stimuli through the sessions.

### Image acquisition and preprocessing

MRI data were acquired with a 3T Siemens Trio scanner equipped with an 8-channel head coil. Head movements during scanning were minimized with the use of padding. Functional data were obtained with a gradient echo-planar imaging (EPI) sequence [2-s repetition time (TR), 30-ms echo time (TE), 192 fields of view, 64 × 64 matrix]. Each volume consisted of 34 axial slices, each with a 4-mm slice thickness with no interslice gap, which resulted in an isotropic voxel size of 3 mm, in an interleaved-descending order. Two high-resolution structural T1-weighted scans were also acquired for normalization images, the localization of activations in the individual and group brains, and the assurance of structural normality. The first scan comprised 34 axial slices collected in the same plane as the EPI images (*TR* = 700 ms, *TE* = 14 ms, voxel dimensions = 1 × 1 × 4 mm). The second was a T1-weighted magnetization-prepared rapid gradient-echo (MP-RAGE) scanning (*TR* = 9.7 ms, *TE* = 4 ms, Flip Angle = 12°, 256 × 256 × 256 isotropic 1-mm voxels). Participants viewed a projection screen from within the magnet bore through a mirror mounted on the head coil. The movements of both eyes were monitored with two infrared cameras. Images from both infrared cameras and a display of visual stimuli were combined in a video frame and recorded onto a DVD. After fMRI acquisition, we checked by visual inspection that participants did not perform pursuit eye movements of the black rotating occluders.

Preprocessing and data analysis were performed with Statistical Parametric Mapping (SPM) 8 software (http://www.fil.ion.ucl.ac.uk/spm/). The first 11 volumes of images corresponding to the first trial in each run were discarded to allow for steady-state magnetization and for the participant state. Therefore, 264 volumes corresponding to 24 trials were analyzed in each run. Preprocessing of the blood oxygenation level-dependent (BOLD) volumes included a slice-time correction with reference to the middle slice acquired in time, the realignment of head motions, the non-linear normalization into the Montreal Neurological Institute (MNI) standard stereotactic space (ICBM152 EPI template) with preserved original voxel size, and spatial smoothing with a 8-mm full-width half-maximum Gaussian kernel. All participants moved no more than 2 mm or 2° in any direction. The spatial non-linear normalization was performed as a two-step procedure. First, a structural image acquired to overlay the EPI images was coregistered to the high-resolution MP-RAGE anatomical structural image. Second, this structural image was spatially normalized. The two resulting transformations were combined into a single transformation and used to spatially normalize the EPI images directly.

### fMRI statistical analysis

Functional data were analyzed with a general linear model (GLM) implemented in SPM8. Statistical analyse s at the individual level were calculated with an event-related design. For each participant, the neural response that was associated with each experimental condition of interest (binding and control) during the maintenance period of each task condition was modeled with a canonical hemodynamic response function. A GLM with regressors was specified for each of the six different conditions (3 task conditions × 2 switch types) during the maintenance period (6.75–15.75 s). Due to the very high accuracy in all conditions, only the correct trials in all of the functional imaging analyses were used. The maintenance period was defined as the period from the beginning of a trial to immediately before the time point of the target change. Each regressor was convolved with the canonical hemodynamic response function included in SPM8. A high-pass filter with a cutoff period of 128 s and an AR (1) model corrected for temporal autocorrelation were applied. The resulting parameter estimates for each regressor at each voxel were then entered into a group analysis in which each participant served as a random effect. Statistical parametric maps of *t*-statistics were thresholded at the significance level of *p* < 0.001, uncorrected for multiple comparisons with a spatial extent threshold of 200 contiguous voxels (Takahama et al., 2010). To identify the maintenance-related brain regions that supported simple or complex feature-location binding, we first compared brain activation in the binding condition with that in the corresponding control condition separately for the three task conditions. In each task condition, a region with maintenance-related activity should exhibit greater activation in the binding condition compared to that in the control condition.

In the next step, in order to compare brain activation between task conditions, region-of-interest (ROI) analyses were implemented for the maintenance period based on the adjusted BOLD signal data from the activation foci peaks identified in the current study and in our previous study (Takahama et al., 2010). In addition, hippocampal involvement has recently been suggested in object-location association in VWM (Duncan et al., 2009). Thus, a ROI was generated in the right hippocampus. To extract the percentage signal changes in the activated regions in each binding and control conditions during the maintenance period, we used the MarsBaR ROI toolbox (Brett et al., 2002). The following ROIs were located in the frontal cortex: (1) right anterior prefrontal cortex (aPFC) (2) right DLPFC (3) right inferior precentral sulcus (infPreCS), and (4) right middle frontal gyrus (MFG), and the following were located in the parietal cortex: (1) right superior parietal lobule (SPL) and (2) right inferior parietal lobule (IPL). In addition, there was a ROI in the right hippocampus. According to Takahama et al. (2010), the signal differences between the binding conditions and the corresponding control conditions in each task were analyzed as a measure of task-relevant feature-location binding-related activity. The activities in the ROIs were evaluated with one-way analyses of variance (ANOVAs) to address the first hypothesis (the complexity of feature-location binding would impact top-down modulation) by testing the main effect of the task conditions (color vs. orientation vs. conjunction). A *post-hoc* test with Tukey's HSD was used to further explore any significant effects revealed by ANOVA.

### Functional connectivity analysis during the maintenance of complex feature-location binding

Based on the univariate analysis results, the 26 contiguous voxels of each participant in the right infPreCS, SPL, and MFG, which were also activated during the spatial updating of simple feature-location binding (Takahama et al., 2010), were used as seed ROIs. To determine the networks with significant functional connectivity between the seed ROIs and the whole brain, the correlations between the single-trial beta parameter estimates were calculated according to the beta-series correlation method (Gazzaley et al., 2004; Rissman et al., 2004). Briefly, raw time-series data were substituted with regression coefficients computed trial-by-trial to assess the correlated variations in amplitude that directly related to the independent variables included in the univariate analysis. For each participant, a new GLM design matrix was constructed to model each trial with a unique covariate, resulting in a total of 216 covariates of interest (24 trials per run × 9 runs) during the maintenance period (6.75–15.75 s). The beta values were then sorted based on switch type (36 control trials and 36 binding trials) separately in the tasks. A correlation coefficient between each ROI and the remaining brain regions was calculated to determine the interactions between the brain regions during the maintenance of task-relevant feature-location binding. Seed correlation maps were created by computing the correlations between the seed beta series (averages across seed voxels) and all brain voxels. The statistical threshold of *p* < 0.005 (two-tailed) with a spatial extent threshold of 15 contiguous voxels was employed for the random effects contrast (Gazzaley et al., 2007). For statistical comparisons, Pearson's *r* values were transformed to Fisher's *z* values. The difference in *z* values between the binding condition and the corresponding control condition in each task was used to measure the connectivity related to task-relevant feature-location binding. The *z* scores were evaluated with One-Way ANOVAs to address the effects of complex feature-location binding (compared to the simple one) on functional connectivity between the seed regions and other brain structures. A *post-hoc* test with Tukey's HSD was used to further explore any significant effects revealed by ANOVA.

### Functional connectivity analysis during the spatial updating of simple feature-location binding

**Figure 2:**
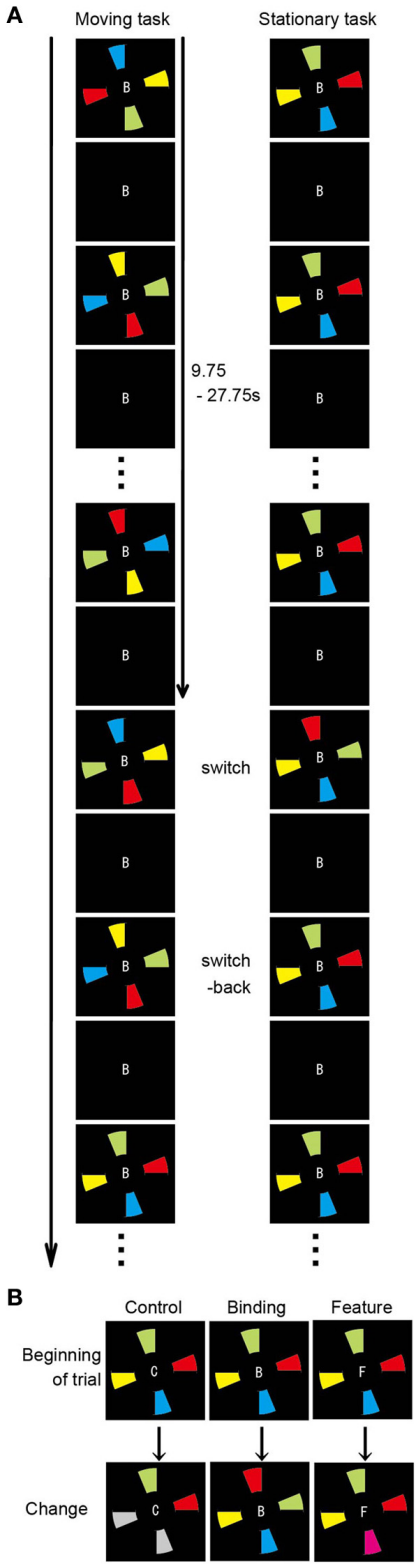
**Overview of the spatial updating task (Takahama et al., 2010). (A)** Schematic diagram of the moving and stationary MOPT tasks. At the beginning of each trial, the occluder and the capitalized letter (B, F, or C) at the center of the visual stimuli are presented for 500 ms. Then, the colored pies (moving task) or the occluder (stationary task) begins to rotate clockwise or anticlockwise. **(B)** Types of target change. In the control condition, 2 of the 4 colored targets turn gray. In the binding condition, the colors are switched for 2 of the 4 targets. In the feature condition, 1 of the 4 colored targets changes to a novel color.

To determine the functional networks that support the spatial updating of simple feature-location binding, we reanalyzed the fMRI data of Takahama et al. (2010) in which the spatial updating of simple feature-location binding was examined, but not that of the above-mentioned experiment. For a complete paradigm description, please refer to Takahama et al. (2010). In brief, a MOPT paradigm in which either differently colored pie-shaped targets or a smoothly rotated black occluder (moving task and stationary task, respectively) was utilized to allow for the spatial updating of simple feature-location binding in VWM (Figure 2). The simple feature-location binding condition in the stationary task was similar to that in the current experiment with the exception that the targets consisted of a single feature (color). The change occurred randomly in each trial. The targets with switched or replaced colors were unpredictable for participants, and the maintenance period varied from trial to trial (9.75–27.75 s). Each task included three change conditions: control, binding, and feature. In the binding condition (2 of 4 colored targets replaced each other), participants monitored a change in simple feature-location binding between objects that were defined by color and location. In the feature condition (1 of 4 colored targets changed to a novel color), participants detected target changes if they maintained just a list of the target colors, but not the location, and matched the presented (perceived) targets to those previously presented (memorized). In the control condition (2 of 4 colored targets turned gray), participants detected target changes even if they did not memorize the target color. Participants were asked to press a response button when they detected a change of the target color. Sixteen trials (eight control, four binding, and four feature trials), each lasting 32 s, were present within each run. The moving and stationary tasks were presented in 10 runs, respectively. Nine of 13 volunteers participated in the above-mentioned experiment. Functional and structural data were acquired with the identical scanner and acquisition parameters. Preprocessing of the imaging data was identical to that in the above-mentioned experiment. The first 16 volumes of images corresponding to the first trial in each run were discarded to allow for steady-state magnetization and for participant state. To identify the functional networks that supported the spatial updating of simple feature-location binding, a GLM design matrix was constructed separately in the moving and stationary tasks to model each trial with a unique covariate during the maintenance period, resulting in a total of 150 covariates of interest (15 trials per run × 10 runs) for each task. For each participant, the neural response associated with each condition of interest (control, binding, and feature) during the maintenance periods of the moving and stationary tasks was separately modeled with a canonical hemodynamic response function. The maintenance period was defined as the period from the trial beginning to immediately before the time point of color change, excluding the first 2 s to eliminate activity related to initial stimulus encoding. The same ROIs used in the above functional connectivity analysis served as the seeds. Because spatial updating was a within-participant factor, the functional connectivity during the maintenance period of the moving task was compared with that of the stationary task. The statistical threshold was *p* < 0.005 (two-tailed) with a spatial extent threshold of 15 contiguous voxels. For statistical comparisons, Pearson's *r* values were transformed to Fisher's *z* values. The differences in *z* values between the experimental (binding and feature) conditions and the corresponding control conditions were used to measure the connectivity related to the maintenance of feature-location binding. The *z* scores were evaluated with Two-Way ANOVA to address the impact of the spatial updating of the simple feature-location binding during the maintenance period.

### Behavioral data analysis

Responses were recorded for each trial to ensure that participants performed the task as instructed. Accuracy was analyzed with Two-Way ANOVA.

## Results

### Behavioral performance

**Figure 3:**
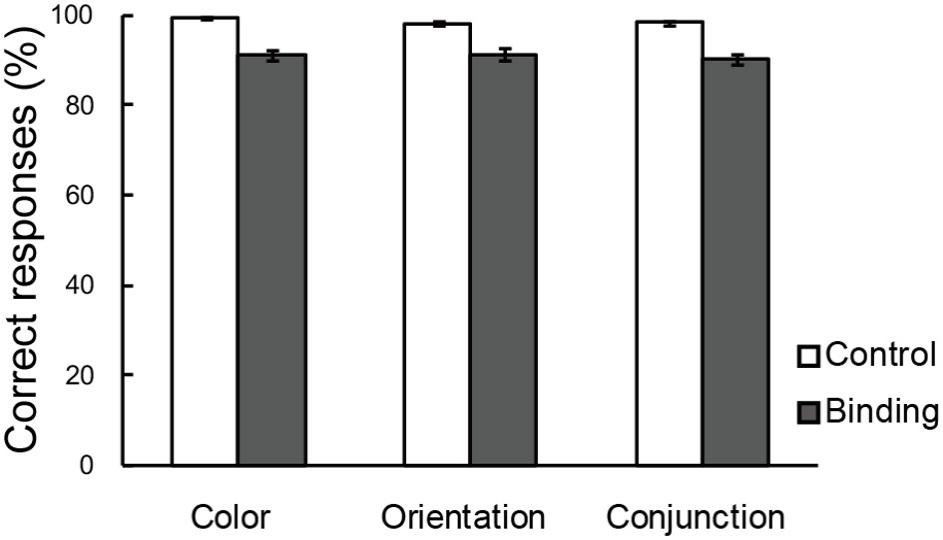
**Correct choices (%) in the change detection tasks**. The error bars represent standard error of the mean (s.e.m).

Figure 3 shows the percentages of correct responses in the color, orientation, and conjunction tasks. Overall, the accuracy was greater than those reported in previous studies that used a MOPT paradigm with multi-dimensional objects (Saiki and Miyatsuji, 2007, 2009), which suggested that the practice sessions prior to the fMRI scanning were sufficient for individuals to reach performance plateau levels, thereby enabling accurate task performance. A repeated-measures ANOVA with a 3 (task condition: color, orientation, or conjunction) × 2 (switch type: control or binding) factorial design revealed a significant main effect for switch type [*F*_(1, 21)_ = 85.05, *p* < 0.01], suggesting that the binding condition was more difficult than the corresponding control condition in each task condition. The interaction between task condition and switch type did not reach statistical significance. It was important to establish that the maintenance-related activities were due to complex feature-location binding processes rather than to task difficulty effects because the pre-training enabled greater performance than what has been observed in previous studies (Saiki and Miyatsuji, 2007, 2009). In addition, there were no differences between the task conditions.

Participants were monitored to ensure that eye movements to track the visual stimuli during the scanning sessions did not take place, and this provided confirmation that imaging results were not attributed to eye movement effects. All of the participants reported that they performed the tasks without verbal encoding of the visual stimuli (targets) during the scanning session as well as during the practice sessions.

### Univariate results for the maintenance of complex feature-location binding

#### Map-wise analysis

**Figure 4:**
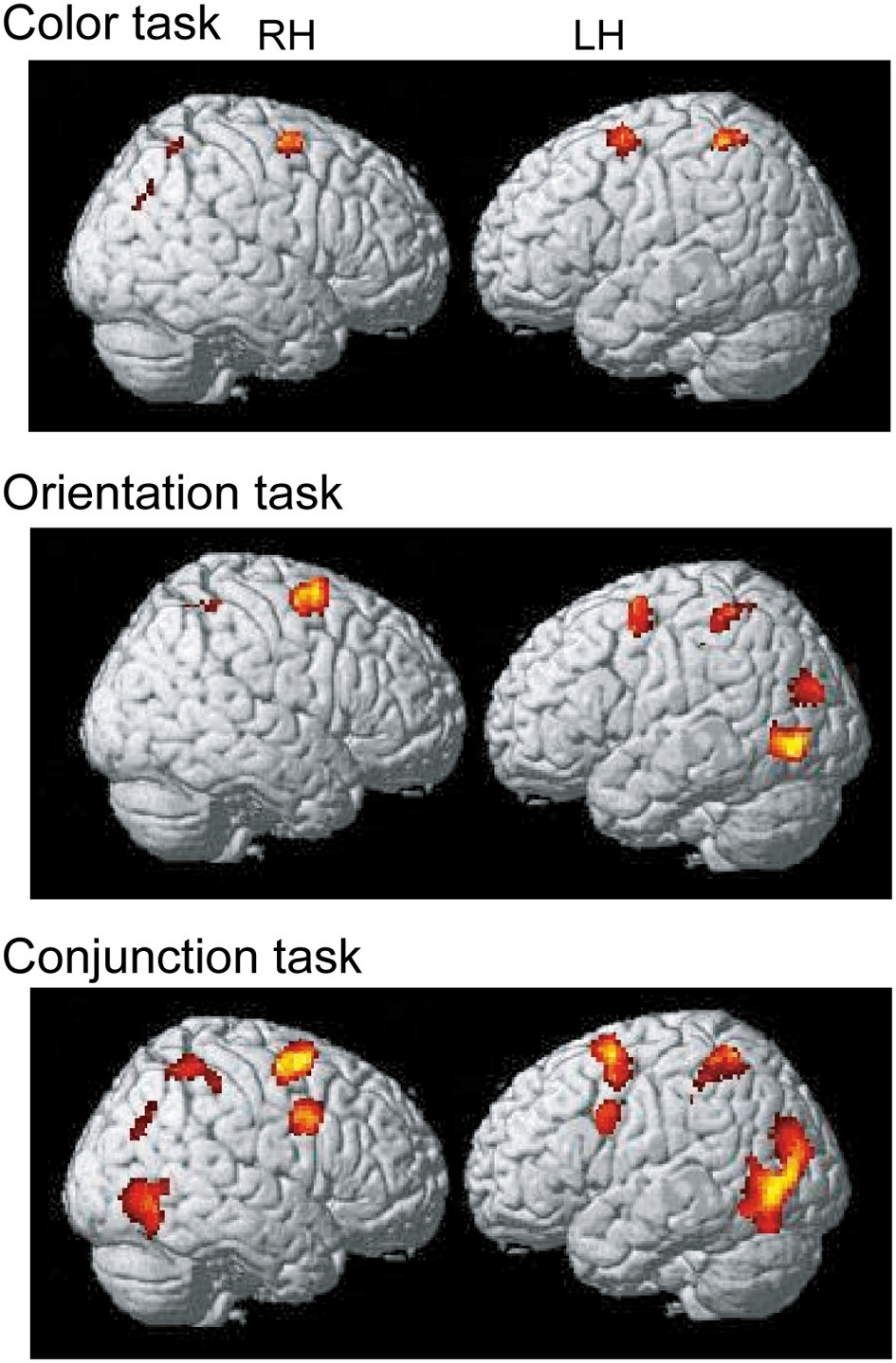
**Statistical parametric map of regions showing greater activation in the binding condition than in the control condition during the maintenance period, displayed on a surface-rendered standard brain**. SPM{t} maps in the color task (upper panel), the orientation task (middle panel), and the conjunction task (bottom panel) (*p* < 0.001, uncorrected, spatial extent threshold of 200 voxels). RH, right hemisphere, LH, left hemisphere.

To identify the maintenance-related brain regions that supported feature-location binding independent of task condition, brain activation in the binding condition was first compared with that in the corresponding control condition in each of the three task conditions. This contrast revealed a number of significantly activated brain regions, including the frontoparietal network, in each task. We predicted that if brain regions were involved in the maintenance of complex feature-location binding driven by matches to top-down modulation, then the activity in those regions would be greater for complex feature-location binding than for simple feature-location binding during the maintenance periods. As shown in Figure 4 and Table 1, regions with maintenance-related activity exhibited greater activation in the binding condition compared to that in the corresponding control condition in each task, and bilateral frontoparietal network activities were greater for the maintenance of complex feature-location binding compared to that for simple feature-location binding. Testing of the opposite contrast, which searched for regions with greater activity in the control compared to the binding conditions, revealed no significant activation in each task. There was no brain activity in the areas related to language processing, including Broca's area, in any of the tasks.

**Table 1:** **MNI coordinates of brain regions that were significantly activated by the binding condition (vs. control condition)**.

		**Cluster**		**Voxel**			
**Brain region**	**BA**	***p*-value**	***k***	***T*-value**	**MNI coordinates**		
**COLOR TASK**							
R Sub-gyral	6	0.125	206	5.45	24	−4	60
R Superior parietal lobule	7	0.033	335	4.45	26	−62	48
**ORIENTATION TASK**							
R Superior frontal gyrus	6	0.013	585	3.93	14	4	68
R Sub-gyral	6			5.25	24	−4	60
R Middle frontal gyrus	6	0.003	863	4.26	24	−8	46
L Inferior parietal lobule	40	0.051	384	3.69	−40	−32	44
L Postcentral gyrus	40			4.49	−32	−40	58
R Precuneus	7	0.002	519	4.32	26	−52	48
**CONJUNCTION TASK**							
R Superior frontal gyrus	8	<0.001	880	4.58	4	16	54
R Sub-gyral	7			6.11	24	−56	56
L Middle frontal gyrus	6	<0.001	998	4.68	−32	2	46
	8			4.57	−28	22	44
	6			4.53	−28	10	68
	6			4.04	−34	4	64
L Inferior frontal gyrus	9			4.93	−42	4	34
L Sub-gyral	6			5.02	−18	−4	54
	6			4.95	−22	−4	56
L Inferior temporal gyrus	19			3.98	−46	−60	−8
R Middle frontal gyrus	8	0.086	244	4.66	4	22	48
R Middle frontal gyrus	6	0.003	609	5.90	32	−2	58
	6			4.71	20	8	64
R Middle frontal gyrus	8	0.04	322	5.16	50	12	40
R Inferior frontal gyrus	9			4.86	40	6	34
L Inferior parietal lobule	40	<0.001	962	3.99	−38	−34	40
L Sub-gyral	7			5.38	−24	−52	54
R Postcentral gyrus	3	<0.001	880	4.03	34	−36	52
	3			3.94	30	−38	48
L Fusiform gyrus	19	<0.001	1421	4.54	−40	−70	−18
R Fusiform gyrus	19			3.59	40	−66	−20
L Middle occipital gyrus	37			5.19	−42	−72	−2
	19			4.91	−46	−80	4
	19			4.74	−40	−84	10
L Middle temporal gyrus	19			4.18	−40	−62	14
L Middle occipital gyrus	19	0.029	356	4.87	−30	−80	20

*The table provides an overview of significantly (p < 0.001, uncorrected, spatial extent threshold of 200 voxels) activated regions related to the maintenance of feature-location binding in the change detection task. P-values are corrected for multiple comparisons at the whole-brain level controlling for familywise error, and k is the number of voxels of each cluster. BA, Brodmann's area; L, left; R, right*.

#### ROI analyses

**Figure 5:**
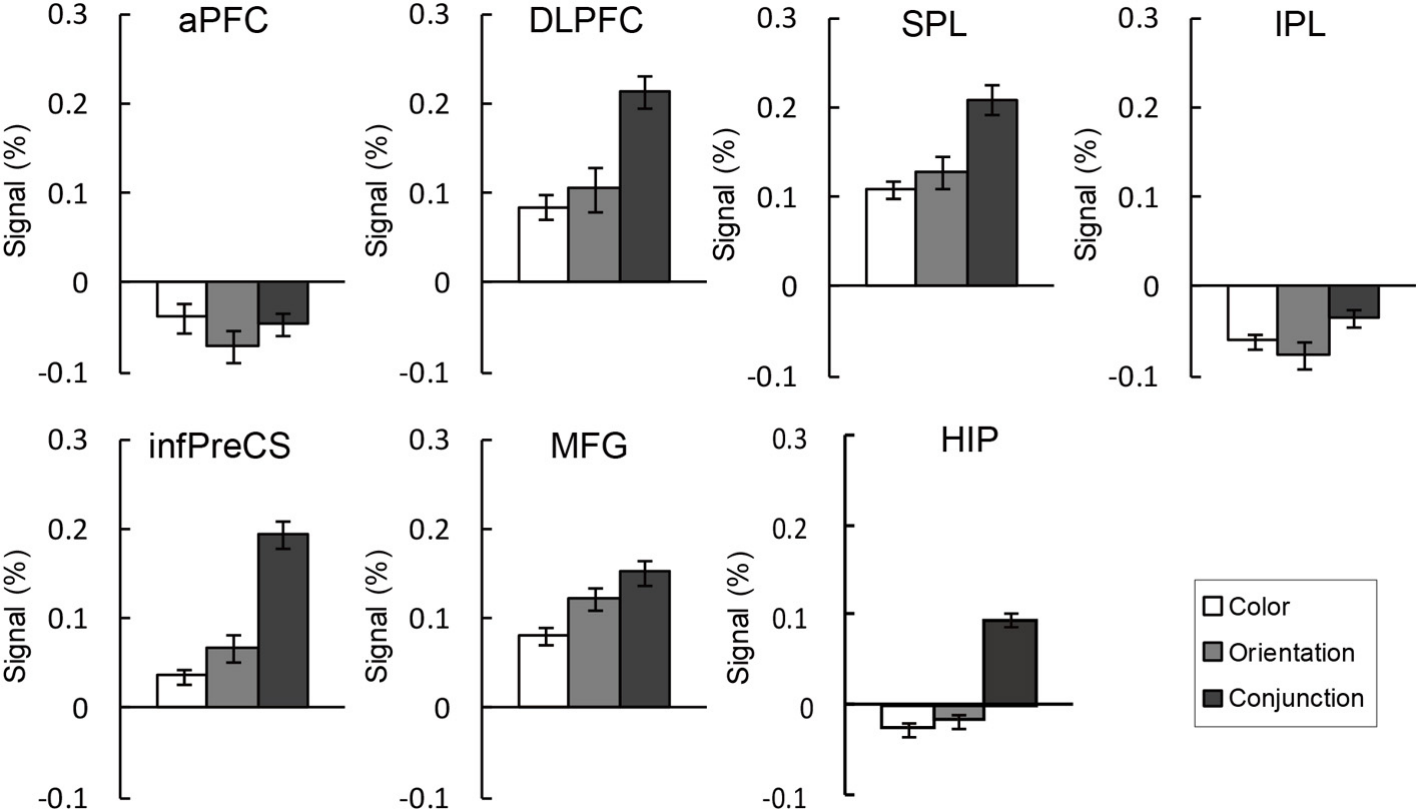
**BOLD signal change (%) extracted ROIs during the maintenance of the binding condition in the change detection tasks**. The error bars represent s.e.m.

ROI analyses (Figure 5) were conducted to identify the specific brain regions involved in the maintenance of complex feature-location binding. First, the signal strengths for each ROI in the binding conditions were compared with those in the control condition in order to identify the regions involved in the activity that was related to the maintenance of feature-location binding. The difference in signal strengths between the binding condition and the corresponding control condition was then used as a measure of the activity that was related to the maintenance of feature-location binding in order to evaluate the effects of complex feature-location binding by comparing the three task conditions.

An ANOVA testing signal change was performed for each ROI. As seen in Figure 5, the right DLPFC, infPreCS, MFG, SPL, and hippocampus exhibited similar patterns. There was a significant main effect of task condition in the right DLPFC, infPreCS, MFG, SPL, and hippocampus [DLPFC: *F*_(2, 42)_ = 3.85, *p* < 0.05; infPreCS: *F*_(2, 42)_ = 7.28, *p* < 0.01; MFG: *F*_(2, 42)_ = 3.30, *p* < 0.05; SPL: *F*_(2, 42)_ = 3.24, *p* < 0.05; hippocampus: *F*_(2, 42)_ = 8.65, *p* < 0.01]. *Post-hoc* analyses revealed that the right DLPFC, infPreCS, SPL, and hippocampus were significantly more activated in the conjunction task than in the color and orientation tasks and that the right MFG was significantly more activated in the conjunction task than in the color task (all *p*s < 0.05). These results indicated that these ROIs were involved in the maintenance of complex feature-location binding. During the maintenance of simple feature-location binding, these ROIs were not statistically more active than in the complex feature-location binding, suggesting that top-down modulation in the monitoring of complex feature-location binding did not suppress neural activity in any brain region. There was no ROI that showed different activity between the color and orientation task.

### Functional connectivity results

#### Network for the maintenance of complex feature-location binding

**Figure 6:**
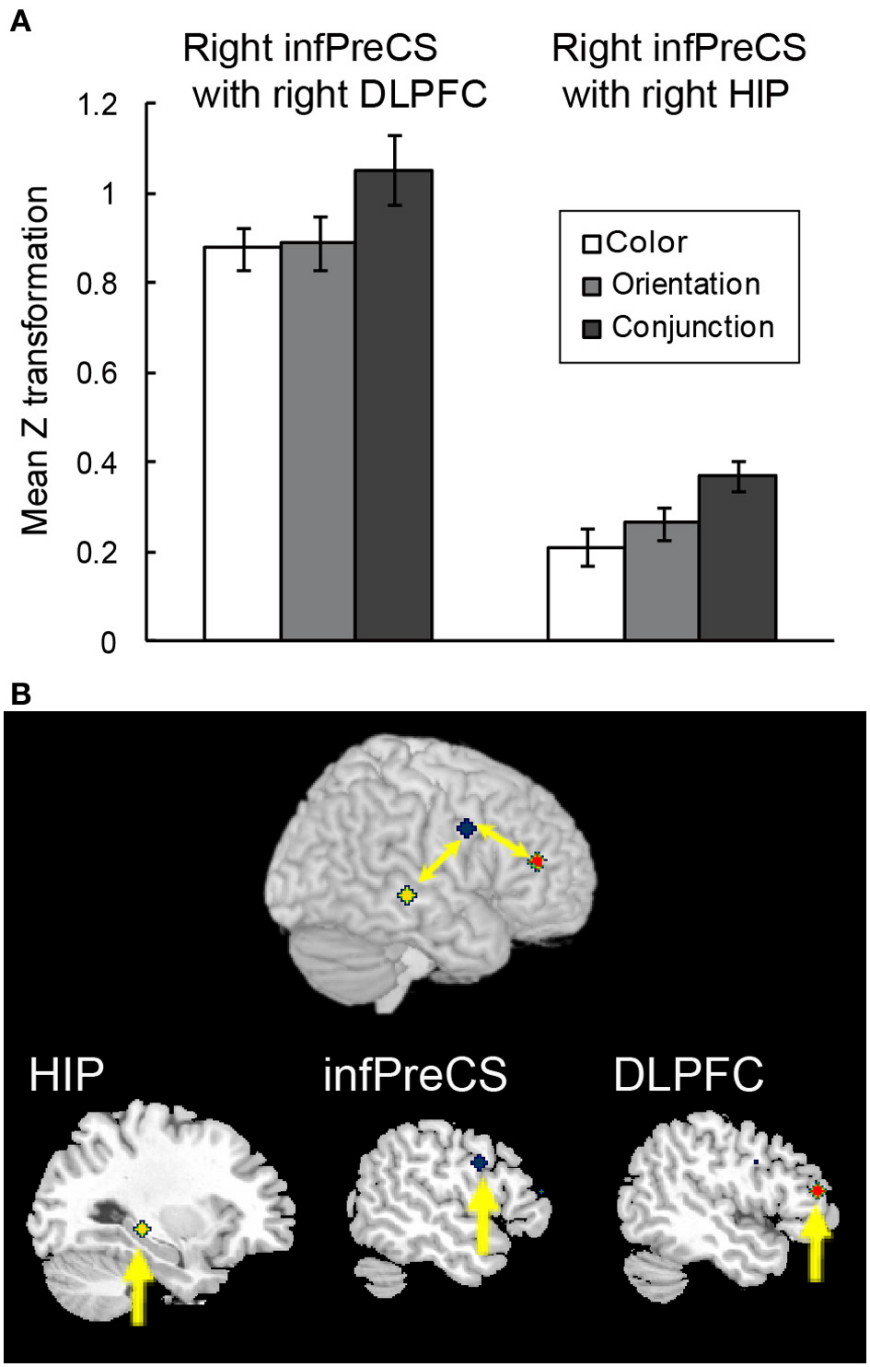
**Functional connectivity during the maintenance of complex feature-location binding. (A)** Functional connectivity between regions is plotted as the mean *z* score transformation of the beta-series correlations during the maintenance of task-relevant feature-location binding. The error bars represent s.e.m. **(B)** A schematic of the complex feature-location binding network; network regions include the right hippocampus (MNI coordinates: 30, −30, −4; yellow cluster), the right infPreCS (MNI coordinates: 52, 2, −32; blue cluster), and the right DLPFC (MNI coordinates: 48, 40, 14; red cluster).

A beta-series correlation analysis was performed to identify the neural networks subserving object representations during the maintenance of complex feature-location binding. The difference in *z* scores between the binding condition and the corresponding control condition was used as a measure of maintenance-related connectivity. Of the three seed ROIs (right infPreCS, SPL, and MFG), the right infPreCS exhibited a significant effect of task condition during the maintenance period, and this correlated with the right DLPFC and hippocampus (Figures 6A,B). One-Way ANOVAs revealed a main effect of task condition in the functional connectivity with the right infPreCS for both regions [right DLPFC: 25 voxels, *F*_(2, 42)_ = 5.13, *p* < 0.05; right hippocampus: 33 voxels, *F*_(2, 42)_ = 7.22, *p* < 0.01]. *Post-hoc* analyses revealed that the functional connectivity with the right infPreCS was greater for the right DLPFC and the hippocampus in the conjunction task compared to those in the color and orientation tasks (all *p*s < 0.05). The right SPL also exhibited a significant effect of task condition on the correlation with the left infPreCS [*F*_(2, 42)_ = 3.61, *p* < 0.05]. A subsequent *post-hoc* analysis revealed greater functional connectivity between the right SPL and left infPreCS in the conjunction task than in the color task (*p* < 0.05). No brain regions were functionally connected to the right MFG.

#### Network for the spatial updating of simple feature-location binding

**Figure 7:**
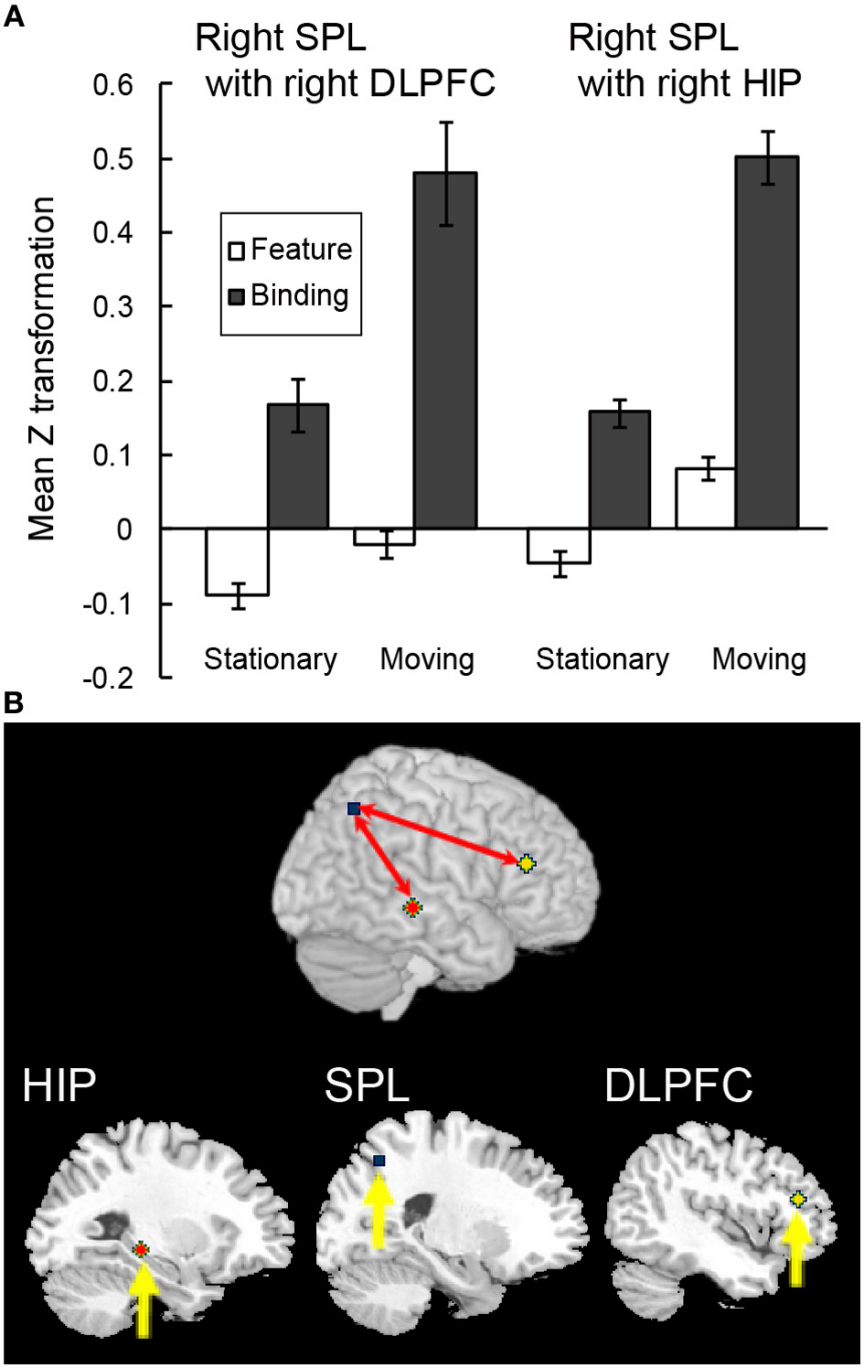
**Functional connectivity during the spatial updating of simple feature-location binding. (A)** Functional connectivity between regions is plotted as the mean *z* score transformation of the beta-series correlations during maintenance of simple feature-location binding in the stationary and moving tasks. Error bars represent s.e.m. **(B)** A schematic of the spatial updating of simple feature-location binding network; network regions include the right hippocampus (MNI coordinates: 28, −30, −6; red cluster), the right SPL (MNI coordinates: 26, −62, 48; blue cluster) and the right DLPFC (MNI coordinates: 44, 32, 18; yellow cluster).

In order to test whether the above-mentioned functional connectivity reflected the characteristics of the maintenance of complex feature-location binding or general memory load in VWM, a functional connectivity analysis during the spatial updating of simple feature-location binding was conducted by using the right infPreCS, SPL, and MFG ROIs as seeds. Of 13 participants, a Smirnov-Grubbs' test for differences in the *z* values between the binding conditions and the control/feature conditions in the moving task identified one outlier (*p* < 0.05), which was removed before performing ANOVAs. Of the three seeds, the right SPL exhibited a significant effect of task condition in the maintenance period, and it correlated with the right DLPFC (33 voxels) and the hippocampus (19 voxels) (Figures 7A,B). For the functional connectivity between the right SPL and DLPFC, a Two-Way ANOVA with a 2 (spatial updating: moving or stationary) × 2 (feature-location binding: binding or feature) design showed main effects of feature-location binding [*F*_(1, 11)_ = 5.27, *p* < 0.05] and spatial updating [*F*_(1, 11)_ = 6.02, *p* < 0.05]. For the functional connectivity between the right SPL and hippocampus, there were significant main effects of binding [*F*_(1, 11)_ = 13.02, *p* < 0.05] and spatial updating [*F*_(1, 11)_ = 6.16, *p* < 0.05]. No significant interaction effects were observed.

It should be noted that the seed ROI during the maintenance of complex feature-location binding differed from that during the spatial updating of simple feature-location binding. However, similar to the functional connectivity during the maintenance of complex feature-location binding, the right DLPFC and hippocampal activity was connected to that of the seed ROIs. These results suggested that, although the right DLPFC and hippocampal activations in the two kinds of feature-location binding tasks were related to memory load, distinct networks were shown to subserve the different aspects of feature-location binding.

## Discussion

The current study identified the brain regions and functional connectivity involved in the selective maintenance of complex feature-location binding with an event-related fMRI design. Through the use of a MOPT paradigm, we manipulated the complexity of feature-location binding that was to be maintained in VWM. Although there was no difference in task performance between complex feature-location binding and simple feature-location binding due to sufficient pre-training, we found brain activities and functional connectivity related to the maintenance of complex feature-location binding. These results possibly imply that the effects of the complexity of feature-location binding result in specific brain activation and functional connectivity. A univariate analysis revealed DLPFC, infPreCS, MFG, SPL, and hippocampal activities during the maintenance of complex feature-location binding compared to simple feature-location binding. There was no brain activity related to the maintenance of specific color-location or orientation-location binding. A functional connectivity analysis indicated that the right DLPFC and hippocampus strongly interacted with the right infPreCS during the maintenance of complex feature-location binding. To test whether the brain activities and functional connectivity related to the maintenance of complex feature-location binding simply reflected general memory load, we examined functional connectivity during the spatial updating of simple feature-location binding by reanalyzing the data of Takahama et al. (2010). Because both the current study and Takahama et al. (2010) utilized the MOPT paradigm, we were able to compare the brain activities and functional connectivity found in the current study with those found in the study by Takahama et al. (2010). The brain regions activated during the maintenance of complex feature-location binding were similar to those activated during the spatial updating of simple feature-location binding, suggesting the broader involvement of these brain regions in feature-location binding. In contrast, a functional connectivity analysis during the spatial updating of simple feature-location binding revealed that the right DLPFC and hippocampus positively interacted with the right SPL. The seed ROI related to the spatial updating of feature-location binding was different from that related to the monitoring of complex feature-location binding, indicating a specific functional interaction for the feature-location binding to be processed, but not general memory load. Taken together, the DLPFC and hippocampus were involved in the maintenance of feature-location binding in general, and the structure of the maintenance network changes depended on the tasks performed with feature-location binding rather than on general memory load.

### Cooperation of the DLPFC and the hippocampus with the frontoparietal network to control feature-location binding

Functional connectivity analyses in a simple VWM task, such as the Sternberg task, have revealed the modulation of connectivity between the DLPFC and hippocampus by memory load (van Vugt et al., 2010). In addition, activities in the prefrontal cortex (inferior frontal gyrus) and hippocampus have been shown to correlate with the fusiform face area activity in a complementary fashion in a delayed face recognition task involving memory load (Rissman et al., 2008). Namely, as the number of to-be-remembered faces increases, the frontal region exhibits a linear decrease in the degree of functional connectivity with the fusiform face area during the delay period, whereas the hippocampus exhibits a linear increase in delay period connectivity with the fusiform face area. The current study examined the functional connectivity during the maintenance of object representation by using a MOPT task that required more complex cognitive control. The results revealed novel aspects of the functional interactions between the DLPFC and hippocampus with a frontoparietal network. First, although the DLPFC and hippocampus modulated the memory load as has been shown by van Vugt et al. (2010), the seed ROI varied depending on the type of feature-location binding that was required by the task. Second, in contrast to Rissman et al. (2008), the current results revealed a cooperative interaction of the DLPFC and hippocampus with the infPreCS in the complexity of feature-location binding and with the SPL in the spatial updating of simple feature-location binding. The results from Rissman et al. (2008) and the current study revealed different aspects of the functional connectivity related to VWM.

Importantly, the regions that exhibited correlated activity with the DLPFC and hippocampus in these MOPT tasks were core regions for the respective cognitive operations. When the maintenance of complex feature-location binding was required, the infPreCS was recruited, whereas the SPL was recruited when spatial updating was required. Maintenance-related activity has been previously reported in the infPreCS (Courtney et al., 1998; Song and Jiang, 2006) close to the junction of the inferior frontal gyrus and the infPreCS (inferior frontal junction area; IFJ). The IFJ is involved in the top-down modulation of information manipulation in VWM (Mohr et al., 2006). The SPL is located near regions that have been shown to be sensitive to VWM capacity in change detection tasks that use objects with simple feature-location binding (Todd and Marois, 2004; Xu and Chun, 2006; labeled superior intraparietal sulcus (IPS) and largely overlaps with our SPL ROI). In addition, the SPL has been shown to be involved in updating rules and stimuli (Montojo and Courtney, 2008) as well as in cognitive control (Tamber-Rosenau et al., 2011). With a multiple-object tracking paradigm (Pylyshyn and Storm, 1988; Scholl and Pylyshyn, 1999) that was used to operate attentional load in a spatial updating situation, previous studies have reported that the SPL is task-sensitive (Culham et al., 2001; Howe et al., 2009) and load-sensitive (Jovicich et al., 2001). Furthermore, increasing activity in the superior parietal gyrus, which is close to our SPL ROI, has been shown to predict increasing activity in the hippocampus in confident memory during encoding in a delayed match-to-sample task of pictures of internal and external scenes that are characterized by multiple objects (Santangelo and Macaluso, 2013). Taken together, these findings suggest that parietotemporal connectivity plays a key role in the integration of the location information of objects during the encoding of VWM.

Therefore, the present results indicated that the frontoparietal network involved in object representation was flexibly connected to the DLPFC and hippocampus depending on the type of operation that was required by a task, suggesting qualitative differences between the two aspects of feature-location binding, but not general memory load. In addition to the previous functional connectivity results that have been found in VWM tasks (Axmacher et al., 2008; Rissman et al., 2008; van Vugt et al., 2010), these findings indicated that the DLPFC and hippocampus constitute core regions of a network that supports different cognitive tasks.

### Brain regions involved in the maintenance of complex feature-location binding

Whole-brain univariate analyses of the fMRI data collected during the maintenance of complex feature-location binding revealed activity in the right infPreCS, MFG, and SPL, and these findings have also been reported in change detection tasks (Linden et al., 2003; Todd and Marois, 2004; Song and Jiang, 2006; Xu and Chun, 2006) and in a MOPT task focusing on the spatial updating of simple feature-location binding (Takahama et al., 2010). In addition, right DLPFC activity has been reported in simple feature-location binding (Prabhakaran et al., 2000), delayed visual discrimination (Linden et al., 2003), manipulation (Mohr et al., 2006; Jackson et al., 2011), and change detection in the spatial updating of simple feature-location binding (Takahama et al., 2010).

In contrast to the dorsal frontoparietal network, the role of the hippocampus in mnemonic binding remains controversial. Some neuroimaging and neuropsychological investigations have shown hippocampal involvement in object-location binding (Olson et al., 2006; Piekema et al., 2006; Hannula and Ranganath, 2008; Duncan et al., 2009) and object-background context binding (Howard et al., 2011), whereas other studies have not reported this (Prabhakaran et al., 2000; Todd and Marois, 2004; Song and Jiang, 2006; Xu and Chun, 2006). The re-analysis of the data from Takahama et al. (2010) in which the hippocampus was included as an ROI revealed hippocampal involvement in the spatial updating of simple feature-location binding in the MOPT task (data not shown) as well as in the maintenance of complex feature-location binding. In addition to the functional connectivity results, the hippocampus might play a role in feature-location binding in a broader context than previously suggested by cooperating with other brain regions.

### Maintenance of selective feature-location binding in the MOPT task

Previous studies have shown the selective processing of feature information in VWM (Song and Jiang, 2006; Xu, 2007). Using a standard change detection task, Song and Jiang (2006) manipulated memory load and the complexity of feature-location binding and have reported load-sensitive activity in the pre-supplementary motor area, frontal eye fields, and inferior frontal sulcus and featural complexity-related activity in the SPL and lateral occipital complex. Xu (2007) has pointed out the involvement of the IPS in the retention of multiple features. However, brain activation related to the maintenance of complex feature-location binding has not yet been described. The successful detection of activity related to complex feature-location binding with the MOPT task was due to the task structure in which target objects gradually appeared and were gradually and repeatedly occluded through the end of the trial. In addition, participants were required to continuously encode, maintain, and retrieve target objects. Unlike change detection tasks that have focused on activity during the blank period, the MOPT task is more sensitive to activity related to complex feature-location binding. However, the MOPT task cannot identify which memory operation (encoding, maintenance, or retrieval) is responsible for this effect. Future studies are necessary to elucidate this issue.

Studies of the manipulation of 1 of 2 properties (color and angle) of two objects in VWM have reported manipulation-specific activity in the dorsal frontoparietal network, including the anterior MFG, the IFJ, and the IPL, which was stronger than that observed for maintenance-related activity (Mohr et al., 2006). Jackson et al. (2011) used the same paradigm and have reported right DLPFC activity during the manipulation of conjunctive binding (computing average color blend and intermediate angle), and their connectivity analyses that used structural equation modeling suggested the importance of a frontoparietal network and a parietal-extrastriate connection but not a hippocampal connection. Although the involvement of similar regions has been previously reported, hippocampal involvement constitutes a critical difference between feature manipulation studies and the current findings. The MOPT task focuses on feature-location binding, whereas feature manipulation tasks emphasize conjunctive binding. In addition, feature manipulation tasks lack visual input during the manipulation, and this could reduce the sensitivity for detecting hippocampal activity. In terms of dealing with object location, the MOPT task might have something in common with VWM tasks showing hippocampal involvement in object-location association (Piekema et al., 2006; Hannula and Ranganath, 2008; Duncan et al., 2009) or object-background context binding (Howard et al., 2011).

### Limitations and the task difficulty of the MOPT task

There was a limitation present in the design of this study. Our results were from 1 MOPT task that included either the maintenance of complex binding or the spatial updating of feature-location binding but not both. The pre-training enabled higher performance in the binding condition than that shown in previous spatial updating tasks (Saiki, 2003; Imaruoka et al., 2005). Although we conducted a pilot experiment comparing complex and simple feature-location binding in the spatial updating condition, the complex feature-location binding in the spatial updating condition was too difficult to compare with the simple feature-location binding even after pre-training (data not shown). Therefore, in the current study, we conducted the complex binding task and spatial updating task separately. Thus, the brain activity and the functional connectivity during the spatial updating of complex binding remain unclear. Additionally, although we did not adopt ROIs in the current study, activities in the right sub-gyral in the color task, the left IPL in the orientation task, and the right MFG in the conjunction task were not significant at cluster level.

## Conclusions

The results of the current study provide evidence for the neural basis and functional connectivity during the maintenance of complex feature-location binding. A univariate analysis during the maintenance of complex feature-location binding revealed activation in a well-known network for maintaining object representations in VWM, which involved the DLPFC, hippocampus, and frontoparietal network, including the infPreCS, MFG, and SPL. The results of the functional connectivity provide evidence for a broader role of the DLPFC and hippocampus in the control of feature-location binding and a selective role of the infPreCS and SPL, which depended on the qualitatively different operations for feature-location binding in VWM.

### Conflict of interest statement

The authors declare that the research was conducted in the absence of any commercial or financial relationships that could be construed as a potential conflict of interest.
